# A roadmap of haustorium morphogenesis in parasitic plants

**DOI:** 10.1093/jxb/erad284

**Published:** 2023-07-24

**Authors:** Gwendolyn K Kirschner, Ting Ting Xiao, Muhammad Jamil, Salim Al-Babili, Vinicius Lube, Ikram Blilou

**Affiliations:** BESE Division, Plant Cell and Developmental Biology, King Abdullah University of Science and Technology, Thuwal, Kingdom of Saudi Arabia; BESE Division, Plant Cell and Developmental Biology, King Abdullah University of Science and Technology, Thuwal, Kingdom of Saudi Arabia; BESE Division, The BioActives Lab, King Abdullah University of Science and Technology, Thuwal, Kingdom of Saudi Arabia; BESE Division, The BioActives Lab, King Abdullah University of Science and Technology, Thuwal, Kingdom of Saudi Arabia; BESE Division, Plant Cell and Developmental Biology, King Abdullah University of Science and Technology, Thuwal, Kingdom of Saudi Arabia; BESE Division, Plant Cell and Developmental Biology, King Abdullah University of Science and Technology, Thuwal, Kingdom of Saudi Arabia; Duke University, USA

**Keywords:** Auxin, *Cuscuta*, cytokinin, haustoria, host, invasion, lateral roots, parasitic plant, *Striga*

## Abstract

Parasitic plants invade their host through their invasive organ, the haustorium. This organ connects to the vasculature of the host roots and hijacks water and nutrients. Although parasitism has evolved independently in plants, haustoria formation follows a similar mechanism throughout different plant species, highlighting the developmental plasticity of plant tissues. Here, we compare three types of haustoria formed by the root and shoot in the plant parasites *Striga* and *Cuscuta*. We discuss mechanisms underlying the interactions with their hosts and how different approaches have contributed to major understanding of haustoria formation and host invasion. We also illustrate the role of auxin and cytokinin in controlling this process.

## Teaser

Parasitic plants invade their host through the formation of haustoria. Here we discuss the development of these organs and highlight the role of the phytohormones auxin and cytokinin.

## Introduction

Unlike animals, where organogenesis takes place during embryogenesis, plants form organs post-embryonically and continue generating new ones throughout their life cycle. This developmental modularity allows plants to adapt to changes in their environment. Parasitic plants invade their host and deprive it of water and nutrients, drastically reducing the host’s fitness and performance, impacting the yield, and causing a severe loss in agricultural fields (Berner *et al.*, 1995; Mishra and Kogan, 2009; Rodenburg *et al.*, 2016). One percent of flowering plants are considered parasitic plants (Westwood *et al.*, 2010). They interact with the host plants by forming a haustorium, a specialized organ used to penetrate the root or shoot tissues of the host, which acts as an interface for the exchange of water and nutrients between the host and parasite (Yoshida *et al.*, 2016).

Parasitic plants can be categorized into different types, including holoparasites, which are unable to perform photosynthesis and are fully dependent on the host to obtain water and nutrients. On the other hand, hemiparasitic plants do not fully depend on the host, because they are photosynthetically active (Westwood *et al.*, 2010).

The best-studied examples of root and shoot parasites are those of the families *Orobanchaceae* and *Convolvulaceae* (Yoshida *et al.*, 2016). Parasites from the *Orobanchaceae* family, including the genera *Orobanche* and *Striga*, invade the root system (Table 1). These plants produce small seeds (between 0.2 mm and 0.5 mm) that can remain in the soil for up to 10 years and germinate when a host is nearby (Musselman, 1980).

**Table 1.** Summary of parasitic plant species mentioned in this review

Plant species	Family	Target, type of parasitism	Host	Haustorium type
*Orobanche* spp. (broomrape)	*Orobanchaceae*	Root, obligate holoparasite (Musselman, 1980)	Tomato, tobacco, potato, hemp, sunflower, peas, lentils (Musselman, 1980)	Terminal haustoria, lateral haustoria (Musselman, 1980; Westwood *et al.*, 2010)
*Striga* spp. (witchweed)	*Orobanchaceae*	Root, obligate hemiparasite (Musselman, 1980)	Maize, sorghum, sugarcane, rice, millet (Musselman, 1980)	Terminal haustoria, lateral haustoria (Musselman, 1980; Westwood *et al.*, 2010)
*Phtheirospermum* ssp.	*Orobanchaceae*	Root, facultative hemiparasite (Ishida *et al.*, 2011)	Medicago, Arabidopsis (Cui *et al.*, 2016; Irving *et al.*, 2019)	Lateral haustoria (Cui *et al.*, 2016)
*Triphysaria versicolor*	*Orobanchaceae*	Root, facultative hemiparasite (Honaas *et al.*, 2019)	Medicago, Arabidopsis, tomato, maize, rice (Honaas *et al.*, 2019)	Lateral haustoria (Matvienko *et al.*, 2001)
*Cuscuta* spp. (dodder)	*Convolvulaceae*	Stem, obligate holoparasite (Kaiser *et al.*, 2015)	Alfalfa, potato, sweet pepper, tomato (Kaiser *et al.*, 2015)	Haustoria derived from stem (Vaughn, 2002)

**Fig. 1.**
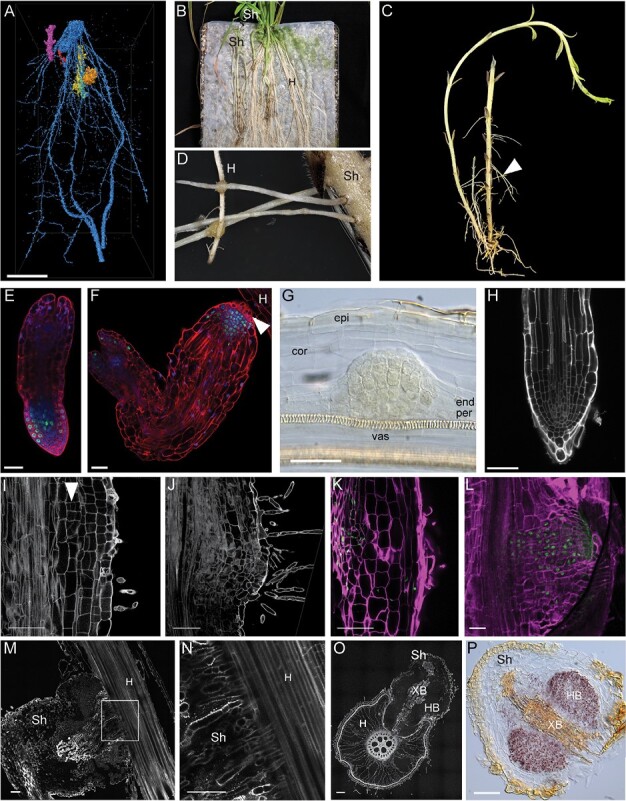
Terminal and lateral haustorium development in *Striga hermonthica*. (A) Micro-CT 3D reconstruction and post-processed image segmentation depicting the association of Striga seedlings (orange, yellow, and purple) attached to the rice roots (blue). (B) Macrophotograph showing Striga plants grown on rice as a host plant on top of nylon mesh. (C) Macrophotograph showing the Striga plant isolated from the host plants. Note the emerging adventitious roots (arrowhead). (D) Attachment of Striga adventitious roots to the host roots by lateral haustoria. (E and F) Confocal image showing the root meristem of Striga seedlings directly after germination (E) and before attachment to the host root (F). Roots were stained with modified pseudo-Schiff propidium iodide (mPS-PI) (red) (Truernit *et al.*, 2008), dividing cells were visualized by 5-ethynyl-2ʹ-deoxyuridine (EdU) staining (green), and nuclei were stained with Hoechst (blue); the arrowhead points to haustorial hairs. (G) Lateral root primordium of Striga roots stained with Lugol, cleared with chloral hydrate, and visualized with Nomarski microscopy. (H) Confocal image of a Striga lateral root tip; cell walls stained with mPS-PI. (I and J) Confocal images of longitudinal vibratome sections of a Striga root developing a lateral haustorium; cell walls and starch granules were stained with mPS-PI; the arrowhead points to periclinal cell divisions in the inner cortex upon haustorium initiation. (K and L) Confocal longitudinal sections showing cell divisions in developing Striga lateral haustoria. EdU-stained nuclei are green and cell walls stained with SCRI Renaissance are in magenta. (M) Longitudinal section of a Striga lateral haustorium during attachment to the host plant; (N) magnification of the area marked in (M). (O) Cross-sections of a Striga lateral haustorium attached to a host root; cell walls stained with mPS-PI. (P) Starch granules accumulation in the Striga lateral haustorium, visualized by Lugol’s staining. cor, cortex; end, endodermis; epi, epidermis; H, host; HB, hyaline body; per, pericycle; Sh, *Striga hermonthica*; vas, vasculature; XB, xylem bridge. Scale bars 20 μm (A, B), 50 μm (E, F, G, H, I, J, K, L, M, N, O, P).

*Striga*, also known as witchweed, is an obligate parasite that grows predominantly in Africa, India, and Southeast Asia and infests crop plants such as maize, sorghum, rice, and millet, causing enormous yield losses (Musselman, 1980). *Striga* seeds germinate only in close proximity to the host roots, as they can sense compounds released from host roots, such as strigolactone. The root of the *Striga* seedling then grows towards the host roots and invades its tissues (Fig. 1A–C) (Musselman, 1980).

The stem parasite *Cuscuta* spp. (Cuscuta) from the *Convolvulaceae* family, also called dodder, is an obligate stem holoparasite that originates from North America and has spread all over the world (Table 1). Cuscuta causes enormous economic loss due to its wide host range, which includes many important crops, such as alfalfa, potato, sweet pepper, and tomato. Most plants of the genus Cuscuta do not have chlorophyll, and therefore completely depend on the host plants (Kaiser *et al.*, 2015). After seed germination, the parasite develops a stem that grows and extends shootward. To maximize the chance of host detection, attachment, and invasion, the Cuscuta stem performs circular movements and, once a host is found, the parasite winds around its stem and forms the haustoria at the attachment sites (Fig.1A, B) (Runyon *et al.*, 2006).

For both parasites, attaching to a host must occur within the first days after germination to ensure their survival, and haustorium formation is vital for a successful invasion. In this review, we aim to highlight developmental programs in both root and shoot parasitic plants, focusing on Striga and Cuscuta as model systems for parasitic plants species. We will discuss their commonalities and differences and how the interplay between the two hormones auxin and cytokinin modulates host invasion, haustorium formation, and establishment in the host plants. We will also compare their developmental programs with those of lateral root initiation and emergence.

## The haustorium as an invasive organ

The haustorium is the organ with which parasitic plants invade their hosts. Two types of haustoria can be recognized based on their origins: the terminal haustorium, which results from the differentiation of the root apical meristem of the parasitic plant and is only found in some obligate parasites, predominantly in the *Orobanchaceae* family; and the lateral haustorium, which can be found in all facultative and some obligate parasites and originates from differentiated tissues of the parasite root or stem (Yoshida *et al.*, 2016). Evolutionarily, lateral haustoria originated first in the transition from non-parasitic to parasitic plants, and only later did the terminal haustoria evolve (Westwood *et al.*, 2010).

### Terminal haustoria: differentiate and invade

Terminal haustoria develop from the differentiation of the root apical meristem a few days after germination. After emerging from the seed coat, the Striga root meristem has a similar structure to that of the model plant Arabidopsis. The meristem contains a quiescent center marked by a low cell division rate; the vasculature cells lay in the center, surrounded by tissue files consisting of one endodermal layer, one to two cortical layers, and one epidermal layer (Fig. 1E) (Xiao *et al.*, 2022). Within 24–48 h after emerging from the seed coat, the roots elongate and, upon sensing the haustorium-inducing factors (HIFs) produced by the host, the Striga root meristem differentiates and forms the terminal haustorium (Hood *et al.*, 1998). During the formation of the terminal haustorium, the cell division rate in the meristem decreases, the meristematic cells differentiate, and root hairs, also termed haustorial hairs, emerge from the epidermis (Fig. 1H) (Xiao *et al.*, 2022). These cells elongate in proximity to the host root and penetrate the host root tissues (Hood *et al.*, 1998). Once they reach the host endodermis, the outermost cells elongate, undergo anticlinal division, and form a palisade arrangement. Then the vascular elements in the haustorium differentiate into xylem elements and establish the vascular connection to the host. After the establishment of the xylem–xylem connection, the cotyledons of the parasite grow out of the seed coat (Hood *et al.*, 1998).

### Lateral haustoria: maximizing invasion

After initial formation of the terminal haustorium as an attachment point to the host plant, the obligate parasites grow out shoots underground, and secondary adventitious roots emerge, from which lateral haustoria can form to enhance the nutrient uptake from the host (Fig. 1B, D, G, H) (Cai *et al.*, 1993). Facultative parasites of the *Orobanchaceae* and the *Scrophulariaceae* families form lateral haustoria that emerge at the root elongation zone, permitting continuous root growth and the formation of multiple haustoria (Matvienko *et al.*, 2001; Ishida *et al.*, 2011; Wakatake *et al.*, 2018).

Lateral haustoria formation involves activation of cell division in multiple root tissue layers, including the pericycle, endodermis, cortex, and epidermis (Figs 1I–L, 3A–C). At the initial stage, anticlinal cell divisions are induced in the epidermis and outer cortex, while periclinal cell divisions are induced in the pericycle, endodermis, and inner cortex (Fig. 1I, K). After the initial anticlinal cell divisions of the epidermis, cells become densely protoplasmic and contain enlarged nuclei, followed by elongation of the cells and emergence of root hairs (Fig. 1J, M, N) (Musselman and Dickison, 1975). Upon attachment to the host, cells continue to divide within the center of the haustorium primordia to form the future vasculature and the hyaline body (Fig. 1L, M, O, P). The hyaline body is the center of the lateral haustorium, consisting of parenchymatic cells with high cytoplasm density and large nuclei, and is proposed to have high metabolic activity and to act as a sink for the host metabolites (Visser *et al.*, 1984; Yoshida *et al.*, 2019).

During terminal and lateral haustoria initiation, starch granules accumulate in the cortical cells of the root (Fig. 1P) (Joel and Losner-Goshen, 1994). Interestingly, no starch granules form during the lateral root formation of the parasitic plant (Fig. 1G). Accumulation of starch granules in the hyaline body supports the idea that the hyaline body in haustoria serves as a host sink tissue (Visser *et al.*, 1984).

### Invading stems: Cuscuta haustoria

**Fig. 2.**
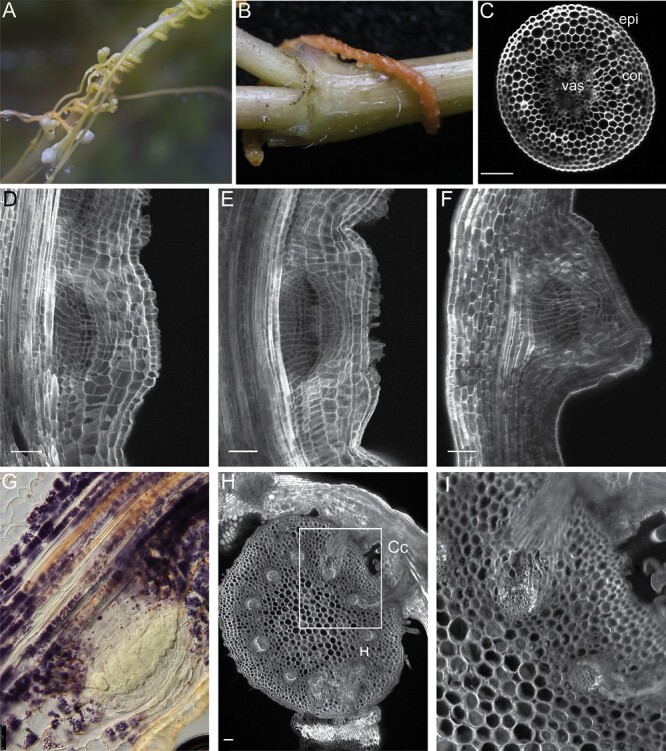
Cuscuta haustorium development. (A and B) Cuscuta plants grown around *Sphagneticola trilobata* as host plants. (C) Confocal image of a vibratome cross-section of a Cuscuta stem; cell walls are stained with SCRI Renaissance. (D–F) Longitudinal vibratome sections of a Cuscuta stem developing a haustorium; cell walls stained are with SCRI Renaissance. (G) Accumulation of starch granules during haustorium formation, visualized by Lugol’s staining. (H and I) Vibratome cross-sections of the host stem with Cuscuta attached to it with haustoria; cell walls stained with mPS-PI (Truernit *et al.*, 2008); (I) magnification of the area marked in (H). Cc, *Cuscuta campestris*; H, host; cor, cortex, epi, epidermis, vas, vasculature; scale bars 50 μm.

After germination, the Cuscuta shoot grows and comes into contact with the host. The Cuscuta shoots twine around the host plant stems and initiate haustorium formation (Fig. 2A, B). The process begins with a swelling of stem areas in close proximity to the host tissue (Fig. 2B) (Vaughn, 2002). Anatomically, Cuscuta stems consist of one layer of epidermis and 6–7 layers of cortex cells surrounding the vasculature in the center (Fig. 2C) (Lee, 2007). At the haustorium initiation, the stems form a plate-like organ, termed the ‘holdfast’, which is formed by anticlinal divisions and elongation of epidermal cells (Fig. 2D, E). The epidermal cells form finger-like structures and develop into unicellular secretory-type trichomes that secrete adhesive compounds (Fig. 2E) (Vaughn, 2002). At the same time, cortex cells divide to create multiple cell layers and contribute to the swelling of the stem (Fig. 2E, G). Then they start to elongate towards the host contact side (Fig. 2F) (Lee, 2007). In the subsequent intrusive phase, the primordium, made up of divided cortex cells, breaks through the outer cortex cells files and the epidermal layer of the parasite, and then through the epidermis of the host and the host cortex (Lee, 2007). The cells at the apex of the haustorium, the so-called ‘searching hyphae’, elongate and search for the vascular tissue of the host plant (Vaughn, 2002). Subsequently, the haustorium develops into conductive vascular cells and the xylic hyphae differentiate into xylem, establishing the haustorial bridge, while the phloic hyphae develop into phloem (Fig. 2G, H) (Vaughn, 2006).

### Similarities and differences between Striga and Cuscuta haustoria formation

**Fig. 3.**
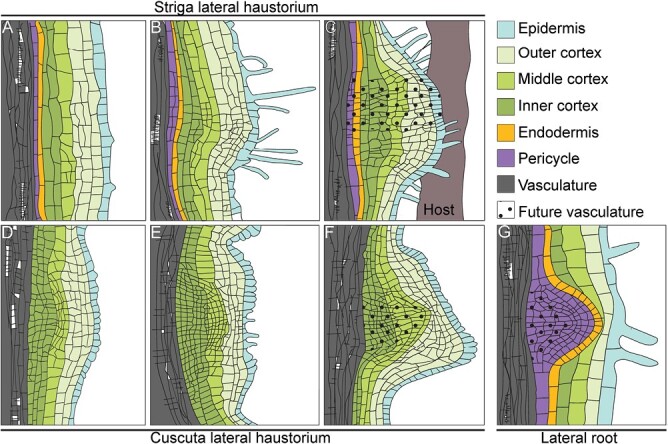
Organogenesis of lateral haustoria and lateral roots. (A–C) Striga lateral haustorium development. (D–F) Cuscuta lateral haustorium development. (G) Lateral root initiation in Arabidopsis; tissues are color-coded according to the key.

Although parasitism within the *Orobanchaceae* and in the *Cuscuta* genus has evolved independently of each other (Westwood *et al.*, 2010), both families deploy a similar mode of host colonization. In both Striga and Cuscuta, haustoria derive from differentiated tissue, either from the root in the case of Striga or from the stem in the case of Cuscuta. The initiation of lateral haustoria in Striga involves cell division within multiple tissues, including the epidermis, cortex, endodermis, and pericycle (Fig. 3A–C). In contrast, in Cuscuta, lateral haustoria formation involves divisions mainly from the cortex (Fig. 3D, E) (Lee, 2007).

Another common feature between root and stem haustorium formation is the remodeling of cell walls by the emerging haustorium. In Cuscuta, genes encoding cell wall-modifying enzymes such as pectin lyases, cellulases, and expansins are up-regulated during the infective stages (Ranjan *et al.*, 2014). In the haustoria, the finger-like epidermal cells secrete cell wall-loosening complexes from Golgi-derived vesicles, which are deposited into the cell wall. The tips of the finger-like cells then bend backward into the cytoplasm, and the cell wall-loosening complexes are prominent in the areas of the cell walls in contact with the host and the areas of infoldings, probably to make the Cuscuta trichomes malleable to form a tight host connection (Vaughn, 2002). Additionally, the secretion of cell wall-loosening enzymes leads to changes in the host cell walls in close proximity to the parasitic haustorium (Johnsen *et al.*, 2015). In Striga, the intrusive cells of the lateral haustoria force their way towards the host vasculature during haustorium invasion of the host tissue. In this process, secretion of substances from the haustorial papillae might play a role in forming an adhesive surface (Neumann *et al.*, 1999). It has been speculated early on that the parasites use enzymatic activity to penetrate between host cortical cells (Musselman and Dickison, 1975). This idea is supported by the up-regulation of catalytic activity-related gene expression in the terminal haustoria during host penetration, with a high proportion of genes categorized as carbohydrate active enzymes (CAZymes) being differentially expressed during the invasion stages of the parasite. These include pectin-degrading enzymes that target primary cell wall components, and proteases (Yoshida *et al.*, 2019). A similar up-regulation of cell wall-related genes was observed in late stages of lateral haustorium development in the facultative parasitic plant *Phtheirospermum japonicum* (Kokla *et al.*, 2022).

Thus, both for stem haustoria and for root-derived haustoria, the remodeling of cell walls by enzymatic activity might have an effect on the parasite cell wall to form an adhesive interaction with the host, as well as on the host cell wall, to facilitate penetration of the haustorium.

### Reprogramming involves recruitment of lateral root developmental modules

Lateral haustoria formation in Striga is initiated by HIFs derived from the host roots. These include 2,6-dimethoxy-*p*-benzoquinone (DMBQ) and its structural analogs (Yoshida *et al.*, 2016). Cuscuta haustoria are induced by the tactile stimulus (i.e. coiling around the host stem) and supplementation with a far-red light stimulus (Bernal-Galeano *et al.*, 2022). HIFs induce a dedifferentiation of root or stem tissue in the parasitic plants, resembling the lateral roots, which originate from the dedifferentiation of the pericycle (Fig. 3G) (Malamy and Benfey, 1997). Indeed, it was found that terminal haustorium development in Striga recruits genes involved in lateral root development (Yoshida *et al.*, 2019). It is plausible that lateral haustorium development uses the same genetic program, given that both terminal and lateral haustoria derive from multiple differentiated cell layers. Similarly, in Cuscuta, haustorium formation recruits genes that are involved in root development in closely related plant species (Sun *et al.*, 2018). The transcriptome during haustorium development in Striga was compared with the genetic program governing lateral root development in Arabidopsis (Yoshida *et al.*, 2019). However, the Arabidopsis lateral root development, originating from the pericycle exclusively, presents an exception rather than the norm, and lateral root formation in other plant species involves more cell files (Xiao *et al.*, 2019). Additionally, lateral root development in Arabidopsis is initiated with anticlinal cell divisions of the pericycle (Malamy and Benfey, 1997), whereas Striga lateral haustoria formation is initiated with periclinal cell divisions in the pericycle, endodermis, and inner cortex (Fig. 1I) (Musselman and Dickison, 1975). Similarly, lateral haustorium development in *P. japonicum* starts with periclinal cell division in the pericycle (Wakatake *et al.*, 2018). In Cuscuta, the cortical cells divide both periclinally and anticlinally for haustorium initiation (Fig. 2D) (Lee, 2007). Striga itself is able to form adventitious roots from the shoot, and higher order lateral roots from them (Fig. 1B, D) (Wolf and Timko, 1991; Cai *et al.*, 1993). Therefore, rather than comparing haustoria with Arabidopsis lateral root formation, it would be more informative to compare the transcriptomal changes during lateral root development and haustorium development in Striga itself to understand the developmental changes. Another module for comparison is the nitrogen-fixing root nodule, which is a lateral root organ formed through the dedifferentiation of pericycle and cortex cells (Xiao *et al*, 2014).

### Auxin and cytokinin balance during haustorium initiation

The balance between phytohormones is important for plant growth, organogenesis, callus induction, and regeneration (i.e. for genetic reprogramming) (Skoog and Miller, 1957; Christianson and Warnick, 1985). Local accumulation of auxin is generally considered as prerequisite for early plant organogenesis (Benková *et al.*, 2003).

Transcriptomic studies in *P. japonicum* showed that haustoria induction by HIFs correlates with the activation of the auxin biosynthesis gene *YUCCA3* in the root epidermis. Furthermore, the reduction of *YUCCA3* activity by gene silencing results in a reduction of haustorium formation (Ishida *et al.*, 2016). In the facultative parasite *Triphysaria versicolor*, exposure to HIFs also leads to auxin accumulation in the root tip, while exogenous application of auxin in addition to HIFs increases the frequency of haustorium formation (Tomilov *et al.*, 2005). In Cuscuta, genes related to polar auxin transport were found to be up-regulated in haustoria (Ranjan *et al.*, 2014). This indicates a functional role for local auxin biosynthesis in lateral haustorium formation. In terminal haustoria of Striga, SOLITARY ROOT (SLR) (INDOLE-3-ACETIC ACID INDUCIBLE 14/IAA14) and AUXIN RESPONSE FACTOR 19 (ARF19) orthologs are specifically expressed in the early stage of haustorium development (Yoshida *et al.*, 2019). In Arabidopsis, these genes work in concert to regulate the expression of an auxin influx carrier that facilitates auxin accumulation during lateral root development (Swarup *et al.*, 2008). Application of auxin to Striga radicles leads to prolonged meristem maintenance with active cell division. In contrast, applying an auxin biosynthesis inhibitor increases the rate of haustoria formation and a cessation of cell division (Xiao *et al.*, 2022). Assuming that terminal and lateral haustorium formation follow a similar mechanism, the increase in auxin concentration at haustorium initiation sites could lead to a reactivation of cell division in the differentiated tissue, which leads to lateral haustorium initiation. However, there is evidence that initiation of haustorium formation between terminal and lateral haustoria actually follows a different pattern: cytokinins trigger the induction of a pre-haustorium in Striga but not in *P. japonicum*, indicating that the processes are influenced by different phytohormone concentrations (Aoki *et al.*, 2022). In *T. versicolor*, the application of cytokinin leads to localized swelling and epidermal hair proliferation near the root tips, resembling the formation of Striga terminal haustoria (Wrobel and Yoder, 2001). Importantly, cytokinin can act as a HIF, hence the application of cytokinin to the parasitic plants would mimic the secretion of HIFs by the host plant (Aoki *et al.*, 2022). Cytokinin, in its role as a HIF, most probably acts through cytokinin receptors in the parasite and partially activates the same transcriptional pathways downstream of the HIF DMBQ (Aoki *et al.*, 2022). A combination of HIFs including cytokinins in host root exudates promotes the expression of cytokinin biosynthesis and signaling genes, leading to an elevated cytokinin concentration in the pre-haustorium, which could further promote haustorium formation (Aoki *et al.*, 2022; Xiao *et al.*, 2022). Cytokinin promotes the formation of the terminal pre-haustorium in Striga and *T. versicolor*, but is not able to induce a lateral haustorium in *P. japonicum*, suggesting that it only acts as a HIF for terminal haustoria. For obligate parasitic plants that form terminal haustoria as the first tool to attach to the host plant, the formation of this organ is crucial for survival. Therefore, it is possible that obligate parasitic plants are sensitive to a broader spectrum of substances that serve as HIFs. Cytokinin signaling reporter lines or knockout mutants of cytokinin pathway components would shed light on how an internal balance of the phytohormones regulates haustorium formation. In summary, the correct concentration of auxin in conjunction with cytokinin and/or other phytohormones is necessary to initiate cell division and de-differentiation of cells involved in the formation of lateral haustoria. Like haustoria, cytokinin and auxin play crucial roles in nodulation. In certain legume species, cytokinin can function as a nodulation factor, inducing nodule formation even in the absence of Rhizobia (Gamas *et al.*, 2017). This shared regulation between parasitic haustoria and nitrogen-fixing root nodules represents yet another common characteristic.

### Parasitic plants manipulate their host by modulating auxin and cytokinin levels

During Striga terminal haustorium development, auxin concentration decreases while cytokinin concentration increases. The reduction of auxin concentration correlates with basal localization of the auxin efflux carriers PIN1 and PIN2 at the epidermis, which suggests that auxin is secreted from the tip of the haustorium to the environment, leading to an increase in auxin response in the host plants (Xiao *et al.*, 2022). Similar processes have been observed for the haustorium development in Cuscuta: during attachment to the host, the content of free IAA in the contact zone increases in both the host and the parasitic tissue (Löffler *et al.*, 1999). This is correlated with an elongation of the haustorial epidermis cells and enlargement of the host cortical cells. It is hypothesized that the auxin is excreted from the haustorium to the host (Löffler *et al.*, 1999). However, it is yet to be determined how the auxin that is produced by the parasite is perceived by the host. The host response will most probably be through the canonical auxin signaling pathway involving the auxin receptors TIR1 and AFB proteins. Recently, it has been shown that nuclear TIR1 mediates slow responses to auxin while AFB1 was found to be important for its rapid response (Chen *et al.*, 2023). With TIR1 and AFB1 having a distinct function in Arabidopsis, it remains to be determined how the parasite modulates the host auxin response, transport, and signaling. The molecular tools that are continuously generated in crops [e.g. reporter lines and clustered regularly interspaced palindromic repeats (CRISPR)/CRISPR-associated protein (Cas) mutants] combined with the advances in imaging technologies, allowing long-term imaging of dynamic interactions (von Wangenheim *et al.*, 2017), will provide a mechanistic understanding on how the parasite impinges on the host roots to secure a successful invasion.

The increase in auxin concentration could lead to cell wall modification in both the haustorium and the host, to form an adhesive surface or facilitate penetration of the host tissue. Auxin is known to regulate the expression of genes encoding cell wall modification enzymes directly (Swarup *et al.*, 2008), and could thereby indirectly influence the cell wall composition, or modify the cell wall directly by acidification of the apoplast, as described by the Acid Growth Theory (Rayle and Cleland, 1992). Evidence for the influence of auxin on changes in cell wall structure was shown recently in lupin, where auxin triggers homogalacturonan demethylesterification in cluster roots and induces the expression of a polygalacturonase in the tissue outside of the outgrowing root primordia, to facilitate outgrowth without damage in the overlying cortex and epidermis cells (Jobert *et al.*, 2022).

Similar to auxin, it was also shown that haustoria are able to manipulate the host phytohormone balance by cytokinin secretion. In *P. japonicum*, genes associated with cytokinin metabolism are up-regulated in the haustorium upon infection, cytokinin levels increase, and cytokinins are transported to host plants via the haustorium (Spallek *et al.*, 2017; Kokla *et al.*, 2022). Cytokinin biosynthesis is likely to take place in the intrusive cells of the *P. japonicum* haustorium, as shown by the expression of the cytokinin biosynthesis gene *PjISOPENTENYLTRANSFERASEa*, and mutation of this gene leads to a loss of cytokinin response in the host roots (Greifenhagen *et al.*, 2021). Cytokinins produced in Cuscuta can also be transferred to host plants and trigger a cytokinin response there (Furuhashi *et al.*, 2014). The cytokinins as mobile signals might induce morphological changes in host roots, such as hypertrophy (i.e. plant tissue overgrowth and an increase in the number of vascular bundles), facilitating tissue penetration of the parasitic plant and formation of a haustorial bridge between parasite and host.

### Controlling phytohormones levels as a strategy against parasitic plant infestation

Strategies to control parasitic plant infestation nowadays comprise the creation of parasite-resistant crop plants (Kaiser *et al.*, 2015; Mutuku *et al.*, 2019), suicidal germination (Kountche *et al.*, 2019), or application of myco-herbicides that target the parasite, but not the host (Rebeka *et al.*, 2013). With the importance of hormone interplay in parasite–host interactions, and with the currently available genomic resources in parasitic plants (Table 2; Yoshida and Kee, 2021), it is important to develop molecular and genetic tools to understand their specific role in this process, to be able to manipulate the interaction to fight parasitic plant infestation. These tools can target both the parasite to make it less infective and the host by increasing its resistance. Application of specific phytohormone signaling inhibitors could prevent haustorium formation and thereby attachment to host plants. Here, a detailed knowledge of the nature of the phytohormone signaling in the parasite is needed to ensure that inhibitors only target processes in the parasite wihout interference with host phytohormones. Conversely, phytohormone signaling components of the host are a potential target, in order to interrupt the tissue adaptations in favor of the parasite. For example, mutants in cytokinin signaling genes in Arabidopsis are resistant to the hypertrophy induced by *P. japonicum* (Spallek *et al.*, 2017). Another possible target is manipulating the cell wall composition of the host to make it inaccessible for cell wall modifications caused by the auxin release of the parasite, and thereby prevent attachment and penetration of the parasite. It has been suggested that the resistance against parasites in host plants is triggered by modifications in the cell wall structure at the infection sites: in Cuscuta-resistant tomato, plant cells walls undergo secondary modifications involving phenylpropanoids and long-chain components that can become cross-linked within the cell wall to prevent infection with the parasite (Kaiser *et al.*, 2015). In Striga-resistant rice cultivars, the resistance is associated with an increase in lignin, and lignin- and glucan-derived compound deposition at the infection site (Mutuku *et al.*, 2019). Interfering with auxin transport, response, and signaling of the parasite, on the other hand, could prevent the auxin-mediated cell wall modifications necessary for the penetration of the haustorium.

**Table 2.** Recent advances in genomes and transcriptomes of the species mentioned in this review

Plant	Genome	Transcriptome
*Striga asiatica*	Yoshida *et al.* (2019)	Yoshida *et al.* (2019)
*Striga hermontica*	Qiu *et al.* (2022)	Yang *et al.* (2015); Wicket *et al.* (2011); Yoshida *et al.* (2019)
*Phtheirospermum japonicum*	Kado and Innan (2018); Yoshida and Kee (2021)	Ogawa *et al.* (2021); Cui *et al.* (2020); Kurotani *et al.* (2020); Ishida *et al.* (2016)
*Triphysaria versicolor*	Not available	Wicket *et al.*, 2011; Yang *et al.*, 2015
*Cuscuta australis*	Sun *et al.*, 2018	Hettenhausen *et al.* (2017)
*Cuscuta campestris*	Vogel *et al.* (2018)	Zhang *et al.* (2021); Kim *et al.* (2014); Shahid *et al.* (2018); Kaga *et al.* (2020)

Genomes and transcriptomes of parasitic plants not mentioned in this study are described in Yoshida and Kee (2021).

The analysis of phytohormonal responses in parasitic plants at the cellular level is still hampered by the lack of suitable molecular biological tools. To analyze phytohormone content in a spatial manner, antibodies against auxin and cytokinin can be used (Xiao *et al.*, 2022); however, they do not allow for monitoring the temporal dynamic of haustoria development *in vivo*. Hairy root transformation in *P. japonicum* represents an important breakthrough to express reporter constructs transiently and create gene knockouts by CRISPR/Cas9; however, no stably transformed reporter lines have been reported to date (Spallek *et al.*, 2017; Greifenhagen *et al.*, 2021).

Although Cuscuta does not develop roots, a protocol for hairy root transformation was recently used to transform the adhesive disk cells of the haustorium. Unfortunately, the transformation events are so far restricted to the adhesive disk, so that analysis of early haustorium formation events by reporter gene expression remains impossible (Lachner *et al.*, 2020).

In conclusion, understanding cellular and molecular mechanisms controlling haustorium formation will remain a challenging task. The availability of genome and transcriptome resources (Table 2; Yoshida and Kee, 2021) and the use of single-cell omics might be promising to deceipher the molecular interplay of *de novo* organogenesis from differentiated tissues. However, it will not be possible, without the development of basic tools for functional biology studies such as a stable transformation protocol, to validate the candidate genes involved in the interaction and communication between the host and the parasite. These tools will also be useful to develop biotechnological approaches that will help in combating parasitic infestations.

## References

[CIT0001] Aoki N , Cui S, Yoshida S. 2022. Cytokinins induce prehaustoria coordinately with quinone signals in the parasitic plant *Striga hermonthica*. Plant and Cell Physiology 63, 1446–1456. 36112485 10.1093/pcp/pcac130

[CIT0002] Benková E , Michniewicz M, Sauer M, Teichmann T, Seifertová D, Jürgens G, Friml J. 2003. Local, efflux-dependent auxin gradients as a common module for plant organ formation. Cell 115, 591–602. 14651850 10.1016/s0092-8674(03)00924-3

[CIT0003] Bernal-Galeano V , Beard K, Westwood JH. 2022. An artificial host system enables the obligate parasite *Cuscuta campestris* to grow and reproduce in vitro. Plant Physiology 189, 687–702. 35294033 10.1093/plphys/kiac106 PMC9157073

[CIT0004] Berner DK , Kling JG, Singh BB. 1995. Striga research and control—a perspective from Africa. Plant Disease 79, 652–660.

[CIT0005] Cai T , Babiker AG, Ejeta G, Butler LG. 1993. Morphological response of witchweed (*Striga asiatica*) to *in vitro* culture. Journal of Experimental Botany 44, 1377–1384.

[CIT0006] Chen H , Li L, Zou M, Qi L, Friml J. 2023. Distinct functions of TIR1 and AFB1 receptors in auxin signalling. Molecular Plant 16, 1117–1119. 37393433 10.1016/j.molp.2023.06.007

[CIT0007] Christianson ML , Warnick DA. 1985. Temporal requirement for phytohormone balance in the control of organogenesis *in vitro*. Developmental Biology 112, 494–497.

[CIT0008] Cui S , Kubota T, Nishiyama T, Juliane K, Shigenobu S, Shibata TF, Toyoda A, Hasebe M, Shirasu K, Yoshida S. 2020. Ethylene signaling mediates host invasion by parasitic plants. Science Advances 6, eabc2385. 33115743 10.1126/sciadv.abc2385 PMC7608805

[CIT0009] Cui S , Wakatake T, Hashimoto K, Saucet SB, Toyooka K, Yoshida S, Shirasu K. 2016. Haustorial hairs are specialized root hairs that support parasitism in the facultative parasitic plant *Phtheirospermum japonicum*. Plant Physiology 170, 1492–1503. 26712864 10.1104/pp.15.01786 PMC4775136

[CIT0010] Furuhashi T , Kojima M, Sakakibara H, Fukushima A, Hirai MY, Furuhashi K. 2014. Morphological and plant hormonal changes during parasitization by *Cuscuta japonica* on *Momordica charantia*. Journal of Plant Interactions 9, 220–232.

[CIT0011] Gamas P , Brault M, Jardinaud MF, Frugier F. 2017. Cytokinins in symbiotic nodulation: when, where, what for? Trends in Plant Science 22, 792–802. 28739135 10.1016/j.tplants.2017.06.012

[CIT0012] Greifenhagen A , Braunstein I, Pfannstiel J, Yoshida S, Shirasu K, Schaller A, Spallek T. 2021. The *Phtheirospermum japonicum* isopentenyltransferase PjIPT1a regulates host cytokinin responses in Arabidopsis. New Phytologist 232, 1582–1590. 34254310 10.1111/nph.17615

[CIT0013] Hettenhausen C , Li J, Zhuang H, et al. 2017. Stem parasitic plant *Cuscuta australis* (dodder) transfers herbivory-induced signals among plants. Proceedings of the National Academy of Sciences, USA 114, E6703–E6709. 10.1073/pnas.1704536114 PMC5559024 28739895

[CIT0014] Honaas LA , Jones S, Farrell N, Kamerow W, Zhang H, Vescio K, Altman NS, Yoder JI, Depamphilis CW. 2019. Risk versus reward: host dependent parasite mortality rates and phenotypes in the facultative generalist *Triphysaria versicolor*. BMC Plant Biology 19, 334. 31370799 10.1186/s12870-019-1856-1 PMC6669981

[CIT0015] Hood ME , Condon JM, Timko MP, Riopel JL. 1998. Primary haustorial development of *Striga asiatica* on host and nonhost species. Phytopathology 88, 70–75. 18945002 10.1094/PHYTO.1998.88.1.70

[CIT0016] Irving LJ , Kim D, Schwier N, Vaughan JKE, Ong G, Hama T. 2019. Host nutrient supply affects the interaction between the hemiparasite *Phtheirospermum japonicum* and its host *Medicago sativa*. Environmental and Experimental Botany 162, 125–132.

[CIT0017] Ishida JK , Wakatake T, Yoshida S, Takebayashi Y, Kasahara H, Wafula E, Depamphilis CW, Namba S, Shirasu K. 2016. Local auxin biosynthesis mediated by a YUCCA flavin monooxygenase regulates haustorium development in the parasitic plant *Phtheirospermum japonicum*. The Plant Cell 28, 1795–1814. 27385817 10.1105/tpc.16.00310 PMC5006708

[CIT0018] Ishida JK , Yoshida S, Ito M, Namba S, Shirasu K. 2011. *Agrobacterium rhizogenes*-mediated transformation of the parasitic plant *Phtheirospermum japonicum*. PLoS One 6, e25802. 21991355 10.1371/journal.pone.0025802 PMC3185032

[CIT0019] Jobert F , Soriano A, Brottier L, Casset C, Divol F, Safran J, Lefebvre V, Pelloux J, Robert S, Péret B. 2022. Auxin triggers pectin modification during rootlet emergence in white lupin. The Plant Journal 112, 1127–1140. 36178138 10.1111/tpj.15993

[CIT0020] Joel DM , Losner-Goshen D. 1994. The attachment organ of the parasitic angiosperms *Orobanche cumana* and *O. aegyptiaca* and its development. Canadian Journal of Botany 72, 564–574.

[CIT0021] Johnsen HR , Striberny B, Olsen S, Vidal-Melgosa S, Fangel JU, Willats WGT, Rose JKC, Krause K. 2015. Cell wall composition profiling of parasitic giant dodder (*Cuscuta reflexa*) and its hosts: *a priori* differences and induced changes. New Phytologist 207, 805–816. 25808919 10.1111/nph.13378

[CIT0022] Kado T , Innan H. 2018. Horizontal gene transfer in five parasite plant species in Orobanchaceae. Genome Biology and Evolution 10, 3196–3210. 30407540 10.1093/gbe/evy219 PMC6294234

[CIT0023] Kaga Y , Yokoyama R, Sano R, Ohtani M, Demura T, Kuroha T, Shinohara N, Nishitani K. 2020. Interspecific signaling between the parasitic plant and the host plants regulate xylem vessel cell differentiation in haustoria of *Cuscuta campestris*. Frontiers in Plant Science 11, 193. 32231674 10.3389/fpls.2020.00193 PMC7082356

[CIT0024] Kaiser B , Vogg G, Fürst UB, Albert M. 2015. Parasitic plants of the genus *Cuscuta* and their interaction with susceptible and resistant host plants. Frontiers in Plant Science 6, 45. 25699071 10.3389/fpls.2015.00045 PMC4316696

[CIT0025] Kim G , LeBlanc ML, Wafula EK, DePamphilis CW, Westwood JH. 2014. Genomic-scale exchange of mRNA between a parasitic plant and its hosts. Science 345, 808–811. 25124438 10.1126/science.1253122

[CIT0026] Kokla A , Leso M, Zhang X, Simura J, Serivichyaswat PT, Cui S, Ljung K, Yoshida S, Melnyk CW. 2022. Nitrogen represses haustoria formation through abscisic acid in the parasitic plant *Phtheirospermum japonicum*. Nature Communications 13, 2976. 10.1038/s41467-022-30550-x PMC9142502 35624089

[CIT0027] Kountche BA , Jamil M, Yonli D, Nikiema MP, Blanco-Ania D, Asami T, Zwanenburg B, Al-Babili S. 2019. Suicidal germination as a control strategy for *Striga hermonthica* (Benth.) in smallholder farms of sub-Saharan Africa. Plants, People, Planet, 1, 107–118.

[CIT0028] Kurotani K , Wakatake T, Ichihashi Y, Okayasu K, Sawai Y, Ogawa S, Cui S, Suzuki T, Shirasu K, Notaguchi M. 2020. Host–parasite tissue adhesion by a secreted type of β-1,4-glucanase in the parasitic plant *Phtheirospermum japonicum*. Communications Biology 3, 407. 32733024 10.1038/s42003-020-01143-5 PMC7393376

[CIT0029] Lachner LAM , Galstyan L, Krause K. 2020. A highly efficient protocol for transforming *Cuscuta reflexa* based on artificially induced infection sites. Plant Direct 4, e00254. 32789286 10.1002/pld3.254 PMC7417715

[CIT0030] Lee KB. 2007. Structure and development of the upper haustorium in the parasitic flowering plant *Cuscuta japonica* (Convolvulaceae). American Journal of Botany 94, 737–745. 21636442 10.3732/ajb.94.5.737

[CIT0031] Löffler C , Czygan FC, Proksch P. 1999. Role of indole-3-acetic acid in the interaction of the phanerogamic parasite Cuscuta and host plants. Plant Biology 1, 613–617.

[CIT0032] Malamy JE , Benfey PN. 1997. Organization and cell differentiation in lateral roots of *Arabidopsis thaliana*. Development 124, 33–44. 9006065 10.1242/dev.124.1.33

[CIT0033] Matvienko M , Torres MJ, Yoder JI. 2001. Transcriptional responses in the hemiparasitic plant *Triphysaria versicolor* to host plant signals. Plant Physiology 127, 272–282. 11553755 10.1104/pp.127.1.272 PMC117983

[CIT0034] Mishra JS , Kogan M. 2009. Biology and management of Cuscuta species. Indian Journal of Weed Science 41, 1–11.

[CIT0035] Musselman LJ. 1980. The biology of Striga, Orobanche, and other root-parasitic weeds. Annual Review of Phytopathology 18, 463–489.

[CIT0036] Musselman LJ , Dickison W. 1975. The structure and development of the haustorium in parasitic Scrophulariaceae. Botanical Journal of the Linnean Society 70, 183–212.

[CIT0037] Mutuku JM , Cui S, Hori C, et al. 2019. The structural integrity of lignin is crucial for resistance against *Striga hermonthica* parasitism in rice. Plant Physiology 179, 1796–1809. 30670602 10.1104/pp.18.01133 PMC6446757

[CIT0038] Neumann U , Vian B, Weber HC, Sallé G. 1999. Interface between haustoria of parasitic members of the Scrophulariaceae and their hosts: a histochemical and immunocytochemical approach. Protoplasma 207, 84–97.

[CIT0039] Ogawa S , Wakatake T, Spallek T, et al. 2021. Subtilase activity in intrusive cells mediates haustorium maturation in parasitic plants. Plant Physiology 185, 1381–1394. 33793894 10.1093/plphys/kiaa001 PMC8133603

[CIT0040] Qiu S , Bradley JM, Zhang P, Chaudhuri R, Blaxter M, Butlin RK, Scholes JD. 2022. Genome-enabled discovery of candidate virulence loci in *Striga hermonthica*, a devastating parasite of African cereal crops. New Phytologist 236, 622–638. 35699626 10.1111/nph.18305 PMC9795911

[CIT0041] Ranjan A , Ichihashi Y, Farhi M, Zumstein K, Townsley B, David-Schwartz R, Sinha NR. 2014. *De novo* assembly and characterization of the transcriptome of the parasitic weed dodder identifies genes associated with plant parasitism. Plant Physiology 166, 1186–1199. 24399359 10.1104/pp.113.234864 PMC4226353

[CIT0042] Rayle DL , Cleland RE. 1992. The acid growth theory of auxin-induced cell elongation is alive and well. Plant Physiology 99, 1271–1274. 11537886 10.1104/pp.99.4.1271 PMC1080619

[CIT0043] Rebeka G , Shimelis H, Laing MD, Tongoona P, Mandefro N. 2013. Evaluation of sorghum genotypes compatibility with *Fusarium oxysporum* under *Striga* infestation. Crop Science 53, 385–393.

[CIT0044] Rodenburg J , Demont M, Zwart SJ, Bastiaans L. 2016. Parasitic weed incidence and related economic losses in rice in Africa. Agriculture, Ecosystems and Environment 235, 306–317.

[CIT0045] Runyon JB , Mescher MC, De Moraes CM. 2006. Volatile chemical cues guide host location and host selection by parasitic plants. Science 313, 1964–1967. 17008532 10.1126/science.1131371

[CIT0046] Shahid S , Kim G, Johnson NR, et al. 2018. MicroRNAs from the parasitic plant *Cuscuta campestris* target host messenger RNAs. Nature 553, 82–85. 29300014 10.1038/nature25027

[CIT0047] Skoog F , Miller CO. 1957. Chemical regulation of growth and organ formation in plant tissues cultured in vitro. Symposia of the Society for Experimental Biology 11, 118–130. 13486467

[CIT0048] Spallek T , Melnyk CW, Wakatake T, Zhang J, Sakamoto Y, Kiba T, Yoshida S, Matsunaga S, Sakakibara H, Shirasu K. 2017. Interspecies hormonal control of host root morphology by parasitic plants. Proceedings of the National Academy of Sciences, USA 114, 5283–5288. 10.1073/pnas.1619078114 PMC5441792 28461500

[CIT0049] Sun G , Xu Y, Liu H, et al. 2018. Large-scale gene losses underlie the genome evolution of parasitic plant *Cuscuta australis*. Nature Communications 9, 4–11. 10.1038/s41467-018-04721-8 PMC6041341 29992948

[CIT0050] Swarup K , Benková E, Swarup R, et al. 2008. The auxin influx carrier LAX3 promotes lateral root emergence. Nature Cell Biology 10, 946–954. 18622388 10.1038/ncb1754

[CIT0051] Tomilov AA , Tomilova NB, Abdallah I, Yoder JI. 2005. Localized hormone fluxes and early haustorium development in the hemiparasitic plant *Triphysaria versicolor*. Plant Physiology 138, 1469–1480. 15965023 10.1104/pp.104.057836 PMC1176418

[CIT0052] Truernit E , Bauby H, Dubreucq B, Grandjean O, Runions J, Barthélémy J, Palauqui J-C. 2008. High-resolution whole-mount imaging of three-dimensional tissue organization and gene expression enables the study of phloem development and structure in Arabidopsis. The Plant Cell 20, 1494–1503. 18523061 10.1105/tpc.107.056069 PMC2483377

[CIT0053] Vaughn KC. 2002. Attachment of the parasitic weed dodder to the host. Protoplasma 219, 227–237. 12099223 10.1007/s007090200024

[CIT0054] Vaughn KC. 2006. Conversion of the searching hyphae of dodder into xylic and phloic hyphae: a cytochemical and immunocytochemical investigation. International Journal of Plant Sciences 167, 1099–1114.

[CIT0055] Visser JH , Dörr I, Kollmann R. 1984. The ‘hyaline body’ of the root parasite *Alectra orobanchoides* benth. (Scrophulariaceae)—its anatomy, ultrastructure and histochemistry. Protoplasma 121, 146–156.

[CIT0056] Vogel A , Schwacke R, Denton AK, et al. 2018. Footprints of parasitism in the genome of the parasitic flowering plant *Cuscuta campestris*. Nature Communications 9, 2515. 10.1038/s41467-018-04344-z PMC6023873 29955043

[CIT0057] von Wangenheim D , Hauschild R, Fendrych M, Barone V, Benková E, Friml J. 2017. Live tracking of moving samples in confocal microscopy for vertically grown roots. eLife 6, e26792. 28628006 10.7554/eLife.26792 PMC5498147

[CIT0058] Wakatake T , Yoshida S, Shirasu K. 2018. Induced cell fate transitions at multiple cell layers configure haustorium development in parasitic plants. Development 145, 1–11. 10.1242/dev.164848 PMC6078332 29950390

[CIT0059] Westwood JH , Yoder JI, Timko MP, DePamphilis CW. 2010. The evolution of parasitism in plants. Trends in Plant Science 15, 227–235. 20153240 10.1016/j.tplants.2010.01.004

[CIT0060] Wickett NJ , Honaas LA, Wafula EK, et al. 2011. Transcriptomes of the parasitic plant family Orobanchaceae reveal surprising conservation of chlorophyll synthesis. Current Biology 21, 2098–2104. 22169535 10.1016/j.cub.2011.11.011

[CIT0061] Wolf SJ , Timko MP. 1991. In vitro root culture: a novel approach to study the obligate parasite *Striga asiatica* (L.) Kuntze. Plant Science 73, 233–242.

[CIT0062] Wrobel RL , Yoder JI. 2001. Differential RNA expression of α-expansin gene family members in the parasitic angiosperm *Triphysaria versicolor* (Scrophulariaceae). Gene 266, 85–93. 11290422 10.1016/s0378-1119(01)00376-6

[CIT0063] Xiao TT , Kirschner GK, Kountche BA, Jamil M, Savina M, Lube V, Mironova V, al Babili S, Blilou I. 2022. A PLETHORA/PIN-FORMED/auxin network mediates prehaustorium formation in the parasitic plant *Striga hermonthica*. Plant Physiology 189, 2281–2297. 35543497 10.1093/plphys/kiac215 PMC9342978

[CIT0064] Xiao TT , Schilderink S, Moling S, Deinum EE, Kondorosi E, Franssen H, Kulikova O, Niebel A, Bisseling T. 2014. Fate map of *Medicago truncatula* root nodules. Development 141, 3517–3528. 25183870 10.1242/dev.110775

[CIT0065] Xiao TT , van Velzen R, Kulikova O, Franken C, Bisseling T. 2019. Lateral root formation involving cell division in both pericycle, cortex and endodermis is a common and ancestral trait in seed plants. Development 146, dev182592. 31591087 10.1242/dev.182592

[CIT0066] Yang Z , Wafula EK, Honaas LA, et al. 2015. Comparative transcriptome analyses reveal core parasitism genes and suggest gene duplication and repurposing as sources of structural novelty. Molecular Biology and Evolution 32, 767–790. 25534030 10.1093/molbev/msu343 PMC4327159

[CIT0067] Yoshida S , Cui S, Ichihashi Y, Shirasu K. 2016. The haustorium, a specialized invasive organ in parasitic plants. Annual Review of Plant Biology 67, 643–667. 10.1146/annurev-arplant-043015-111702 27128469

[CIT0068] Yoshida S , Kee YJ. 2021. Large-scale sequencing paves the way for genomic and genetic analyses in parasitic plants. Current Opinion in Biotechnology 70, 248–254. 34242992 10.1016/j.copbio.2021.06.011

[CIT0069] Yoshida S , Kim S, Wafula EK, et al. 2019. Genome sequence of *Striga asiatica* provides insight into the evolution of plant parasitism. Current Biology 29, 3041–3052. 31522940 10.1016/j.cub.2019.07.086

[CIT0070] Zhang J , Xu Y, Xie J, Zhuang H, Liu H, Shen G, Wu J. 2021. Parasite dodder enables transfer of bidirectional systemic nitrogen signals between host plants. Plant Physiology 185, 1395–1410. 33793912 10.1093/plphys/kiaa004 PMC8133666

